# Warmed, humidified CO_2_ insufflation benefits intraoperative core temperature during laparoscopic surgery: A meta‐analysis

**DOI:** 10.1111/ases.12350

**Published:** 2016-12-14

**Authors:** Meara Dean, Robert Ramsay, Alexander Heriot, John Mackay, Richard Hiscock, A. Craig Lynch

**Affiliations:** ^1^ Epworth HealthCareMelbourneVictoriaAustralia; ^2^ Division of Cancer SurgeryPeter MacCallum Cancer CentreMelbourneVictoriaAustralia; ^3^ Sir Peter MacCallum Department of OncologyUniversity of MelbourneMelbourneVictoriaAustralia; ^4^ Department of Surgery, St Vincent's HospitalUniversity of MelbourneMelbourneVictoriaAustralia

**Keywords:** Laparoscopy, meta‐analysis, temperature

## Abstract

**Background:**

Intraoperative hypothermia is linked to postoperative adverse events. The use of warmed, humidified CO_2_ to establish pneumoperitoneum during laparoscopy has been associated with reduced incidence of intraoperative hypothermia. However, the small number and variable quality of published studies have caused uncertainty about the potential benefit of this therapy. This meta‐analysis was conducted to specifically evaluate the effects of warmed, humidified CO_2_ during laparoscopy.

**Methods:**

An electronic database search identified randomized controlled trials performed on adults who underwent laparoscopic abdominal surgery under general anesthesia with either warmed, humidified CO_2_ or cold, dry CO_2_. The main outcome measure of interest was change in intraoperative core body temperature.

**Results:**

The database search identified 320 studies as potentially relevant, and of these, 13 met the inclusion criteria and were included in the analysis. During laparoscopic surgery, use of warmed, humidified CO_2_ is associated with a significant increase in intraoperative core temperature (mean temperature change, 0.3°C), when compared with cold, dry CO_2_ insufflation_._

**Conclusion:**

Warmed, humidified CO_2_ insufflation during laparoscopic abdominal surgery has been demonstrated to improve intraoperative maintenance of normothermia when compared with cold, dry CO_2._

## Introduction

Intraoperative hypothermia is common in open and minimally invasive abdominal surgery, with up to 20% of patients experiencing unintended perioperative hypothermia (defined as a core temperature below 36°C) 1. Adverse events are associated with even mild decreases in intraoperative core temperature and include myocardial ischemia and arrhythmias, changes in blood coagulation, increased blood loss and transfusion requirements, prolonged recovery time, and increased septic complications and mortality 2, 3, 4, 5, 6, 7, 8.

Active warming methods used to prevent intraoperative hypothermia include forced air systems; warmed, humidified ventilator circuits; and warmed intravenous and irrigation fluids. By avoiding an open wound with exposed viscera, laparoscopic surgery is expected to cause less hypothermia than open surgery. Several animal studies have reported decreased perioperative hypothermia with the use of warmed, humidified CO_2_ with the effect more apparent during longer procedures. 9, 10, 11, 12. However, the current literature suggests that rates of hypothermia are equivalent in laparoscopic and open surgery as reported in clinical studies 13, 14.

The mechanism underpinning heat loss during laparoscopic CO_2_ insufflation has been studied by Bessel *et al.* in a paired pig model 15. They showed that the minimal temperature change in pigs receiving warmed, humidified CO_2_ was similar to pigs undergoing anesthesia alone. However, a three‐hour administration of either cool or heated dry gas resulted in a temperature drop of 1.3–1.7°C. This precipitous temperature drop was attributed to the heat energy required to achieve water saturation of the cold gas plus equilibrating its temperature; the heat energy was 20 times greater than the small amount of energy required to heat dry CO_2_ gas to 37°C.

The aim of this meta‐analysis is to evaluate the effect of warmed, humidified CO_2_ insufflation on intraoperative core temperature to build a consensus across multiple randomized controlled trials.

## Materials and Methods

Relevant studies were identified that compared the core intraoperative temperature of patients over 18 years of age who received insufflation with standard cold, dry CO_2_ and warmed, humidified CO_2_. In all studies, patients underwent laparoscopy under general anesthesia, with or without the use of an external warming blanket.

### Search strategy

**Figure 1:**
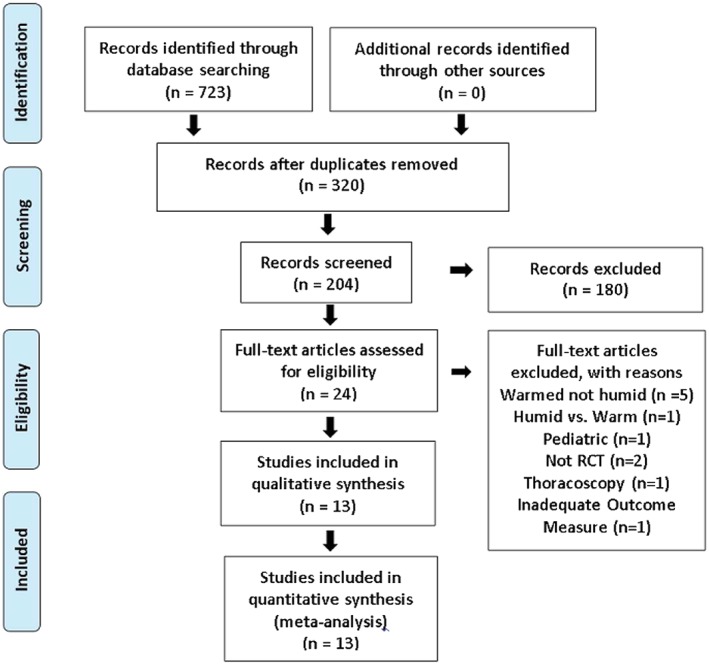
Study selection using Preferred Reporting Items for Systematic Review and Meta‐Analysis 2009 flow diagram. RCT, randomized controlled trial.

A systemic review of published works was conducted according to the Preferred Reporting Items for Systematic Review and Meta‐Analysis guidelines (Figure 1). Searches were limited to human studies without language restrictions. The search terms “humidified” and “humidification” were used in combination with “insufflation,” “carbon dioxide,” “CO_2,_” “laparoscopy,” and “minimally invasive surgery.” Additionally, there were further searches combining these terms and the most commonly performed laparoscopic operations. Reference lists were searched manually to identify further relevant articles.

### Validity assessment

Assessment of the randomized controlled trials was performed by one author blinded to journal title, article title, and publication authorship. Methodological quality was determined using the Jadad scale. Extracted data were verified by another author.

### Statistical analysis

Data from eligible studies were collected into an Excel data spreadsheet (Microsoft, Redmond, USA) and analyzed using Stata statistical software (StataCorp, College States, USA) and the Metan package 16. Publication bias was assessed using Egger's small studies test and contour enhanced funnel plots, both part of the Metan package.

Data extracted for each of the study groups included mean ± SD temperature difference (end of case minus start of case), preoperative mean ± SD temperature, postoperative mean ± SD temperature, number of participants, duration of surgery in minutes, and use of an intraoperative warming blanket. In the absence of reported difference SD, we chose to use those from the Cochrane meta‐analysis by Birch *et al.* (warmed, humidified gas = 0.52, dry gas = 0.66) based upon the largest difference SD for experimental and control groups where difference SD were recorded 17. We also performed a sensitivity analysis using studies in which the mean temperature difference and difference SD were presented. Random effects meta‐analysis was used to determine overall pooled estimates, with results presented using Forest plots. Significance level was set at 0.05. Heterogeneity was assessed using the *Q* and *I* ^2^ statistics. Predetermined subgroup analysis based on the use of an external warming device and operation length was performed.

## Results

A total of 723 papers were identified and entered into a database; 403 were duplicates (same study title appearing in different search results), leaving 320 potentially relevant publications. Thirteen studies met the inclusion criteria and were included in the meta‐analysis (Figure 1) 18, 19, 20, 21, 22, 23, 24, 25, 26, 27, 28, 29, 30. They comprised a total of 796 patients. Studies were published from 1998 to 2015 and included between 20 and 150 participants. There were four studies on laparoscopic gynecological procedures, three on laparoscopic cholecystectomy, four on laparoscopic gastric bypass, two on laparoscopic fundoplication, and one on laparoscopic colonic resection (Table 1).

**Table 1:** Study summary table

Study	Randomized	Double‐blinded	Adequate control	Jadad score	Procedure	Humidified(*n*)	Control (*n*)	Site	External warming device	Operating time (min)‡
Ott *et al.* 18	Yes†	No	Yes	1	Gynecological laparoscopy	36	36	Endotracheal	No	38–262
Mouton *et al.* 19	Yes†	No	Yes	2	Laparoscopic cholecystectomy	20	20	Esophageal	Not specified	40
Nguyen *et al.* 20	Yes†	No	Yes	1	Laparoscopic fundoplication	10	10	Esophageal	Yes	107 ± 12
Farley *et al.* 21	Yes†	Yes†	Yes	5	Laparoscopic cholecystectomy	49	52	Esophageal	Not specified	91 ± 23
Kissler *et al.* 22	Yes	Yes	Yes	5	Gynecological laparoscopy	17	19	Intravesical	Not specified	62 ± 30
Savel *et al.* 23	Yes†	Yes†	Yes	1	Laparoscopic Roux‐en‐Y gastric bypass	15	15	Esophageal	Not specified	76 ± 16
Hamza *et al.* 24	Yes	Yes	Yes	5	Laparoscopic Roux‐en‐Y gastric bypass	23	21	Esophageal	No	120 ± 24
Champion & Williams 25	Yes†	Yes†	Yes	0	Laparoscopic Roux‐en‐Y gastric bypass	25	25	Rectal	No	62 ± 10
Davis *et al.* 26	Yes	Yes†	Yes	1	Laparoscopic Roux‐en‐Y gastric bypass	11	11	Intravesical	No	84
Manwaring *et al.* 27	Yes	Yes	Yes	5	Gynecological laparoscopy	30	30	Not specified	Yes	50 ± 17
Sammour *et al.* 28	Yes	Yes	Yes	5	Laparoscopic colonic resection	35	39	Esophageal	Yes	176 ± 49
Agaev *et al.* 29	Yes†	Yes†	Yes	1	Laparoscopic fundoplication or cholecystectomy	66	84	Not specified	Not specified	Intervention = 42 Control = 56
Herrmann & De Wilde 30	Yes	Yes	Yes	5	Laparoscopy‐assisted vaginal hysterectomy	48	49	Intranasal probe	Yes	86 ± 29

† Inadequately described.

‡ Mean ± SD or range.

**Figure 2:**
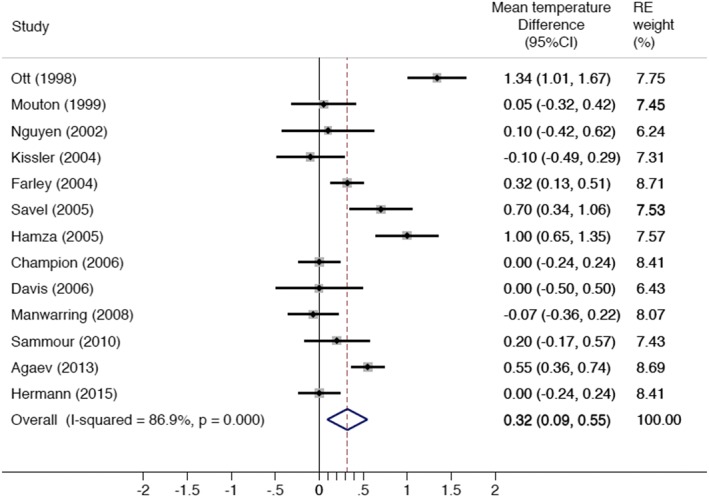
Overall core body temperature change. CI, confidence interval; RE, random effects.

Overall there was a significant difference in mean core temperature change between the warmed, humidified group and the cold, dry group, with an effect size of +0.3°C (95% confidence interval [CI]: 0.1–0.6) (Table 2, Figure 2). Both the *Q* statistic (91.6, 12 degrees of freedom, associated *P* <0.0001) and *I* ^2^ statistic (87%) indicated significant heterogeneity between the studies.

**Table 2:** Preoperative and postoperative temperature data used in the random effects meta‐analysis for heated humidified and dry gas

Study	Year	Heated humidified gas			Dry gas		
		Number	Mean difference	SD difference	Number	Mean difference	SD difference
Ott *et al.* 18	1998	25	−0.3	0.52	25	−1.6	0.66
Mouton *et al.* 19	1999	20	−0.3	0.52	20	−0.3	0.66
Nguyen *et al.* 20	2002	10	0.4	0.52	10	0.3	0.66
Farley *et al.* 21	2004	49	0.3	0.60	52	−0.0	0.30
Kissler *et al.* 22	2004	17	−0.5	0.52	19	−0.4	0.66
Savel *et al.* 23	2005	15	0.4	0.50	15	−0.3	0.50
Hamza *et al.* 24	2005	23	−0.7	0.52	21	−1.7	0.66
Champion & Williams 25	2006	25	−0.4	0.40	25	−0.4	0.45
Davis *et al.* 26	2006	11	0.4	0.52	11	0.4	0.66
Manwaring *et al.* 27	2008	30	−0.2	0.52	30	−0.1	0.61
Sammour *et al.* 28	2010	35	0.6	0.90	39	0.4	0.70
Agaev *et al.* 29	2013	66	0.5	0.52	84	−0.1	0.66
Herrmann & De Wilde 30	2015	48	−0.1	0.52	49	−0.1	0.66

**Figure 3:**
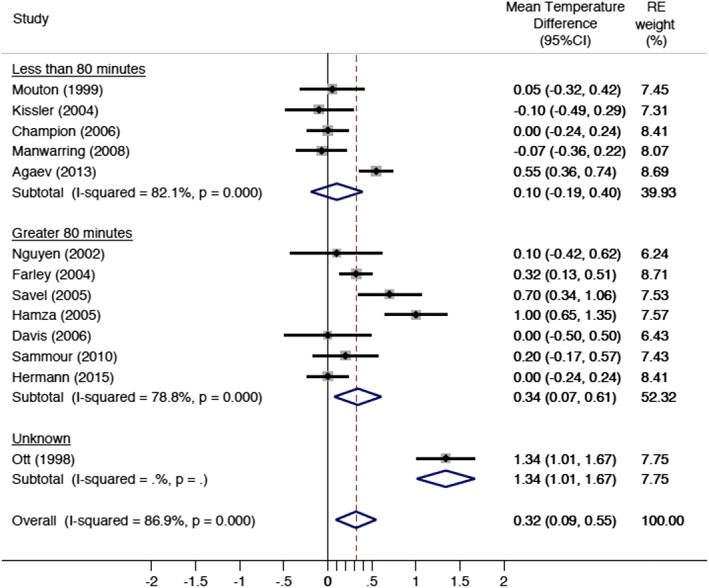
Overall core body temperature change by use of external warming blanket. CI, confidence interval; RE, random effects.

Subset analysis was performed for the use of an intraoperative external warming blanket and duration of operation (<80 min vs >80 min). Among the four studies that documented the use of an intraoperative external warming blanket, there was no intergroup difference in intraoperative temperature change (Figure 3), with a random effects mean difference of 0.02°C (95%CI: −0.13–0.18). In the studies in which no external warming blanket was used, there was an increase in mean intraoperative temperature of 0.59°C (95%CI: −0.12–1.30), but this did not reach significance.

**Figure 4:**
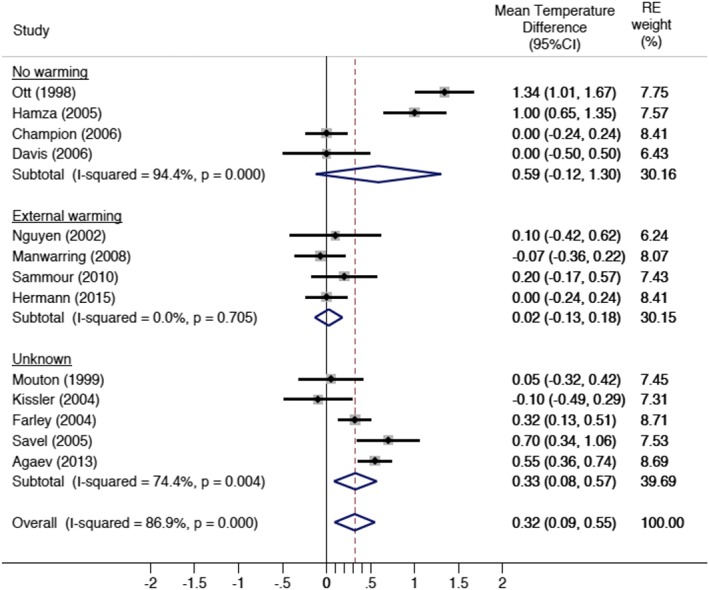
Overall core body temperature change by operation duration. CI, confidence interval; RE, random effects.

When data were stratified by duration of surgery (Figure 4), the studies with a mean duration of surgery greater than 80 min showed a significant improvement in mean temperature with warmed, humidified CO_2_ of 0.34°C (95%CI: 0.07–0.61). There was no intergroup temperature difference in studies with a mean duration of surgery less than 80 minutes (mean difference, 0.1; 95%CI: −0.2–0.4). Only three studies reported the difference SD for treatment groups with a mean difference SD of 0.67°C for the heated humidified group and 0.53°C for the dry gas group 21, 27, 28. Meta‐analysis both overall and for these two subgroups (<80 min vs >80 min) using these SD values for the 10 studies did not substantively change the results.

## Discussion

The effects of perioperative hypothermia are well recognized; it is now standard of care to warm surgical patients. The UK National Institute for Health and Care Excellence guideline on hypothermia prevention and management in adults having surgery provides advice on preventing hypothermia at each stage of the perioperative pathway (preoperative, intraoperative, and postoperative) 31. Likewise, the US Institute for Healthcare Improvement suggests the use of active methods to maintain normothermia in surgical patients 32. However, even when active warming techniques are used, perioperative hypothermia is common 33. This meta‐analysis demonstrates a benefit with the use of warmed, humidified CO_2_, with an overall effect size of +0.3°C. The significance of this result should be considered against the heterogeneity of included studies—namely, the method of core temperature measurement and recording, the types of surgery, patient group characteristics, and the use of external warming. This meta‐analysis is in agreement with a similar study by Balayssac *et al.* 34. Based on a slightly different search strategy, they showed a similar increase in perioperative temperature with warmed, humidified CO_2_; their meta‐analysis included one study using warming alone 35.

### Method of temperature measurement

Thermoregulatory vasoconstriction maintains a temperature gradient between the core and the periphery of 2°C to 4°C 8. During abdominal surgery, the most commonly used method of temperature measurement is the esophageal temperature probe. This method is cost‐effective, carries low patient risk, and provides an accurate measure of thermal status 36. Ideally, the probe should be inserted to about 40 cm into the esophagus; the more proximal probe, the higher the probability that the precision of the probe will be compromised due to cooling by respiratory gases, which leads to an inaccurate temperature measurement 37. Esophageal measurement is considered to be more precise than rectal temperature, which lags behind core temperature, and bladder temperature, which is dependent on urine flow 38, 39.

The included studies varied in the methods used to measure temperature (esophageal, endotracheal, intravesical, nasal, rectal, nasal) and the interval of temperature change recorded (start/end of surgery, start/end of insufflation, start of surgery/recovery). The measure used by most studies is successive 15‐min temperatures added to obtain an average temperature and an overall temperature change. Although the lack of independence and autocorrelation between successive temperature measurements is rarely taken into account in such studies, this is an area where accuracy of recorded temperature differences can be improved in future research. Calculating the area under the temperature curve is an alternative to using repeated measures based upon anova, and this should be considered in study design.

### External warming

The importance of patient prewarming in the prevention of intraoperative hypothermia has been realized in recent years 40. Nevertheless, prewarming was not described in any of the included studies. Indeed, the use of active external patient warming was inconsistently reported by the included studies. Four studies described the application of active external warming blankets to all patients in both treatment and control groups. Three of these studies stated explicitly that active warming was used, but none documented the temperature setting used 20, 27, 28. Subgroup analysis found no difference in core temperature change in these four studies and when compared to the temperature change see in the studies in which external warming was either not used or not documented, supporting the use of an external warmed air device to reduce intraoperative hypothermia.

### Type of surgery and patient characteristics

The greatest heat loss occurs in the first hour post‐induction; the inhibition of vasoconstriction has the effect of core‐to‐peripheral redistribution of body heat, decreasing core temperature by 1.0–1.5°C, even in actively warmed patients 36, 41. After this redistribution of core heat is complete, it is apparent that core temperature progressively increases 33. Studies that aim to assess perioperative heat loss should therefore ideally involve assessing patients undergoing surgery with operating times exceeding 1 h.

Patient characteristics such as body habitus are also known to affect thermoregulation. For instance, bariatric patients tend to experience reduced intraoperative heat loss because of insulation provided by adipose tissue 42. Four studies in this meta‐analysis included bariatric patients. Other patient characteristics, such as age, coexisting medical conditions, active infection, and medications, can affect thermoregulation, were not controlled, and often were not reported in the included studies 41.

Additionally, operating room temperature and humidity affect perioperative hypothermia. Operating temperatures are typically kept below 23°C for the comfort of staff, but this temperature setting contributes to patient hypothermia. Few studies reported the operating room temperature settings, and no studies included data on this variable. Moreover, hypothermia is more common in patients having spinal or epidural anesthetics because of impaired central and peripheral thermoregulation 8. The use of regional anesthesia was not reported by the included studies, and such data should be documented in future studies.

### Meta‐analysis technique

The Cochrane systematic review by Birch *et al.* reported no significant difference in temperature change with the use of warmed, humidified CO_2_ 17. Importantly, the meta‐analysis presented here identified and corrected several errors in the Cochrane analysis (Champion and Williams, incorrect temperature value for control group; Sammour *et al.*, incorrect temperature change values; Farley *et al.*, incorrect standard deviation; Mouton *et al.*, incorrect study group size) and included two additional recently published studies 19, 21, 25, 28, 29, 39. For the 10 studies in which difference SD were not presented (i.e. all except Farley *et al.*, Manwarring *et al.*, and Herrmann and De Wilde), we have presented the analysis using the difference SD employed by Birch *et al.* 17. Analysis using difference SD based upon the three studies with recorded difference SD, both overall and for the two subgroup analyses, led to the same conclusions.

### Other potential considerations for the use of warmed, humidified gas insufflation

Cold, dry CO_2_ insufflation is associated with detrimental local effects on the peritoneum, including peritoneal surface desiccation, inflammation, and acidification 10, 43. In contrast, several studies have reported that the use of warmed, humidified CO_2_ for insufflation reduces peritoneal damage through reduced desiccation and the concomitant inflammatory response 10, 44, 45. The consequent tissue trauma caused by CO_2_ insufflation appears to offer a favorable environment for the attachment and implantation of viable tumor cells. In animal studies, there was significantly greater intraperitoneal tumor growth with cold, dry gas than in cohorts either without any insufflation or with insufflation with warmed, humidified CO_2_ 46, 47, 48.

Optimal perioperative thermal homeostasis is an important component of sound surgical practice. The risks of hypothermia are known and have been numerously documented. Prevention of hypothermia reduces length of hospital stay, extent of intensive care unit admissions, and magnitude of postoperative morbidity and mortality. The peritoneal physiological environment created by warmed, humidified CO_2_ affords no known risks while imposing minimal additional equipment and cost. This meta‐analysis indicates that the use of warmed, humidified CO_2_ provides improved temperature control across a spectrum of surgical settings and thus should be considered as a positive adjunct to laparoscopic surgery.
